# Uncertainty Quantification in SAR Induced by Ultra-High-Field MRI RF Coil via High-Dimensional Model Representation

**DOI:** 10.3390/bioengineering11070730

**Published:** 2024-07-18

**Authors:** Xi Wang, Shao Ying Huang, Abdulkadir C. Yucel

**Affiliations:** 1 School of Electrical and Electronic Engineering, Nanyang Technological University, Singapore 639798, Singapore; xi002@e.ntu.edu.sg; 2 Engineering Product Development Department, Singapore University of Technology and Design, Singapore 487372, Singapore; huangshaoying@sutd.edu.sg

**Keywords:** generalized polynomial chaos (gPC), high-dimensional model representation (HDMR), magnetic resonance imaging (MRI), MRI safety, sensitivity analysis, surrogate model, ultra-high-field (UHF) MRI, uncertainty quantification

## Abstract

As magnetic field strength in Magnetic Resonance Imaging (MRI) technology increases, maintaining the specific absorption rate (SAR) within safe limits across human head tissues becomes challenging due to the formation of standing waves at a shortened wavelength. Compounding this challenge is the uncertainty in the dielectric properties of head tissues, which notably affects the SAR induced by the radiofrequency (RF) coils in an ultra-high-field (UHF) MRI system. To this end, this study introduces a computational framework to quantify the impacts of uncertainties in head tissues’ dielectric properties on the induced SAR. The framework employs a surrogate model-assisted Monte Carlo (MC) technique, efficiently generating surrogate models of MRI observables (electric fields and SAR) and utilizing them to compute SAR statistics. Particularly, the framework leverages a high-dimensional model representation technique, which constructs the surrogate models of the MRI observables via univariate and bivariate component functions, approximated through generalized polynomial chaos expansions. The numerical results demonstrate the efficiency of the proposed technique, requiring significantly fewer deterministic simulations compared with traditional MC methods and other surrogate model-assisted MC techniques utilizing machine learning algorithms, all while maintaining high accuracy in SAR statistics. Specifically, the proposed framework constructs surrogate models of a local SAR with an average relative error of 0.28% using 289 simulations, outperforming the machine learning-based surrogate modeling techniques considered in this study. Furthermore, the SAR statistics obtained by the proposed framework reveal fluctuations of up to 30% in SAR values within specific head regions. These findings highlight the critical importance of considering dielectric property uncertainties to ensure MRI safety, particularly in 7 T MRI systems.

## 1. Introduction

Magnetic Resonance Imaging (MRI) stands as a cornerstone in medical diagnostics, offering unparalleled detail in imaging soft tissues without the risks associated with ionizing radiation [1,2]. During MRI scans, subjects are exposed to strong magnetic fields and radiofrequency (RF) pulses. These RF pulses cause hydrogen atoms in the body to resonate, emitting fields captured by the RF coil to produce high-resolution images of tissues and organs [2]. However, this process results in energy deposition in the body, quantified via the specific absorption rate (SAR), which measures the RF energy absorbed per unit mass during the scans [3]. High SAR values indicate elevated tissue temperatures, potentially causing tissue damage and burns. Consequently, ensuring SAR levels remain within safe limits is paramount for subject safety during an MRI scan [4]. Nonetheless, this task becomes challenging with the increasing frequency of the RF pulses in the latest ultra-high-field (UHF) MRI systems, where standing waves form at a shortened wavelength [5,6].

The UHF MRI systems, particularly those operating at 7 T, have become available for clinical applications. The first 7 T MRI system was introduced to the market in 2017 [7,8]. As of 2023, over 100 7 T MRI systems are in operation globally [9]. These machines utilize a stronger magnetic field than their predecessors, offering enhanced image clarity and resolution but presenting new challenges in assessing SAR values and safety considerations. The increased magnetic field corresponds to an increased frequency of RF pulses, resulting in non-uniform electric fields (E-fields) and SAR distributions within the body [7,8]. Given these advancements, understanding the SAR distributions in the context of 7 T MRI systems has become important. Furthermore, the variability in SAR distributions is influenced by various factors beyond just the non-uniformity of E-fields. Among these factors, the dielectric properties of the tissues have a significant impact on the amount of RF energy absorbed [5,6,10]. Values of the dielectric properties, permittivity and conductivity, are not fixed but exhibit ±20% variability around their nominal values due to the change in physiological parameters, e.g., oxygen levels [11,12,13,14,15,16], reflecting the natural heterogeneity found within biological tissues. Such variability (or uncertainty) in dielectric properties can directly lead to variations in SAR values, which significantly impact image quality and patient safety. These variations in the SAR can introduce artifacts and reduce image clarity, thus compromising diagnostic accuracy and potentially necessitating repeat scans [17]. Additionally, such variations may cause tissue heating and thermal injuries in patients. This variability can also introduce significant discrepancies between the actual and computed E-field and SAR distributions [18,19]. Therefore, to lower such discrepancies and ensure the SAR values comply with high-tier SAR limit standards [20,21], there is a need for computational frameworks that can accurately quantify and assess the impact of uncertainties in tissue dielectric properties on the induced SAR values.

Current tools for computing SAR distributions rely on deterministic electromagnetic (EM) simulators [22,23]. However, these simulators do not account for the effects of uncertainties in tissue dielectric properties while generating the simulation results. In addressing this need, the traditional Monte Carlo (MC) methods [24,25] can be applied in conjunction with these simulators. The traditional MC methods require the execution of the deterministic simulator for numerous randomly selected realizations of tissue dielectric properties, yielding statistical measures, such as the mean and standard deviation, of the SAR. Despite their straightforward implementation, MC methods are inefficient as they require a large number of deterministic simulations, each of which is computationally expensive for EM characterization in MRI scenarios [24]. To overcome these challenges, surrogate model-assisted MC presents itself as a viable alternative [26]. This approach leverages surrogate models to approximate the outcomes of deterministic simulators, thereby significantly reducing the computational burden associated with traditional MC methods [26]. By integrating surrogate models, the surrogate model-assisted MC method retains the versatility of traditional MC approaches while acquiring statistics more efficiently.

Previously, researchers have successfully utilized surrogate modeling techniques and surrogate model-assisted MC methods to quantify the effects of uncertainties in various bioelectromagnetic problems, including transcranial magnetic stimulation (TMS), transcranial direct current stimulation (tDCS), and cellphone radiation safety assessment studies. For TMS, non-intrusive generalized polynomial chaos (gPC) [19] and high-dimensional model representation (HDMR) techniques [18] have been applied in uncertainty quantification. Similarly, in tDCS, various techniques, including the non-intrusive stochastic collocation method (SCM) [27] and gPC [19], have been implemented to achieve the same objective. Furthermore, both gPC and SCM [28,29] have been employed to quantify the uncertainties in the SAR induced due to cellphone radiation. These applications have yielded promising results in terms of accuracy and efficiency, and shown the necessity and effectiveness of the surrogate model-assisted MC methods for uncertainty quantification in bioelectromagnetic problems. However, to our knowledge, no study has performed uncertainty quantification in the SAR induced by UHF MRI RF coils and necessarily employed these surrogate model-assisted MC methods.

This paper proposes a surrogate model-assisted MC framework to quantify the effects of uncertain tissue dielectric properties on the SAR induced by UHF MRI RF coils. The framework initially generates surrogate models of E-fields by using the outcomes of a small number of deterministic simulations performed by MARIE [23], an open-source MRI EM analysis software. Then, the SAR’s mean, standard deviation, and sensitivity indices are obtained via MC using the surrogate models instead of the deterministic EM simulator. Specifically, the proposed framework constructs surrogate models by leveraging the HDMR technique [30], combined with the gPC method, which requires significantly fewer deterministic simulations than the traditional MC method’s brute-force application [30,31,32]. The HDMR technique allows approximation of the multivariate MRI observables (E-field and SAR) via univariate and bivariate component functions. Doing so effectively tackles the ‘curse of dimensionality’ arising during surrogate model generation of multivariate functions. While the technique iteratively includes the most significant component functions (of most influential tissue dielectric parameters) in the HDMR expansion, it approximates each component function via the gPC method [31,32].

The numerical results (in Section 3) show that the proposed HDMR-based technique demonstrated superior performance over other surrogate modeling methods, including random vector functional link (RVFL) [33,34], extreme learning machine (ELM) [35,36], single-layer neural network (NN) [36], Gaussian process (GP) [37], and least square-based gPC [38]. Among all these techniques, the HDMR required the minimum number of simulations while providing the statistics with the highest accuracy, thanks to its capability of selectively incorporating the most significant component functions (with combined effects of dielectric properties).

The contributions of this study are threefold:

- This is the first and foremost study performing uncertainty quantification of the SAR induced by UHF MRI RF coils. It demonstrates the significance of uncertainties in the dielectric properties of human head tissues, which can cause up to 30% fluctuations in SAR values within specific head regions, as demonstrated in the numerical results section.
- This study proposes an HDMR-based surrogate modeling technique, which emerges as the best among various tested surrogate modeling methods for approximating E-fields and SAR induced by UHF MRI RF coils. The technique obtains the surrogate models with a mean relative error of 0.28% by only 289 deterministic simulations, surpassing the accuracy and efficiency of other surrogate modeling methods, as shown in the numerical results section.
- Finally, this study conducts statistical and sensitivity analyses on SAR values. The statistical analysis presents theoretical maximum 1g-SAR and 10g-SAR values after incorporating the uncertainties in tissue dielectric properties, which underscores their importance in MRI safety assessment. Furthermore, the sensitivity analysis shows the uncertainties in which tissues’ dielectric properties affect the SAR values more in certain regions of the brain.

The rest of this paper is organized as follows. Section 2 presents the preliminary concepts, the formulation of the proposed gPC-based HDMR technique, and the general information on the deterministic simulator MARIE used in this study. Section 3 provides numerical results and analysis, focusing on the accuracy and efficiency of the proposed framework and presenting statistical and sensitivity analyses. Finally, Section 4 presents the conclusion, summarizing the study’s key findings.

## 2. Formulation and Methods

Please refer to Appendix A for the nomenclature, which lists mathematical terms used throughout the paper.

### 2.1. Preliminary Concepts

Throughout this study, the dielectric properties of six human head tissues are assumed to be uncertain. Specifically, these uncertain parameters are the relative permittivity, $\varepsilon_{r}$, and conductivity, σ, of white matter, grey matter, cerebrospinal fluid, bone, scalp, and eye humor, with nominal values provided in Table 1, such that there exist N=12 uncertain parameters in total. The uncertain parameters are represented by random variables, $x^{k}$, k=1,2,…,N, each uniformly distributed over a finite 1-D random domain defined by the ranges $\left[a^{k},b^{k}\right]$, as outlined in Table 1. These random variables, $x^{k}$, k=1,2,…,N, assumed to be mutually independent, are integrated into a 12-dimensional vector, denoted as x$=\left[x^{1},x^{2},\ldots ,x^{N-1},x^{N}\right]=\left[\varepsilon_{r1},\ldots ,\varepsilon_{r6},\sigma_{1},\ldots ,\sigma_{6}\right]$, where each symbol and corresponding random variable in x are provided in Table 1 [11,12,13,14,15,16]. Given the absence of prior knowledge regarding the distributions of input parameters, they are assumed to follow uniform distributions according to the principle of maximum entropy and vary ±20% around their nominal values [11,12,13,14,15,16,39,40]. Moreover, while this study primarily focuses on the uncertainties associated with tissue properties, any uncertain parameter deemed significant for SAR variation can be incorporated in x.

Let y=F(x) denote a vector storing the observable values, and *F* represent a complex and nonlinear function evaluated by a deterministic simulator for a given input vector, x. Specifically, in this study, the vector $y=[y^{1},\ldots ,y^{N_{\mathrm{vox}}}]$, with a dimension of $N_{\mathrm{vox}}$ = 889,850, contains the values of MRI observables (E-fields or SAR) on the voxels, where $N_{\mathrm{vox}}$ is the number of voxels used to discretize the tissues in the voxelized head model. While the observables are selected as E-fields and SAR in this study, the framework is applicable to any MRI observable deemed important.

The traditional MC method [24,25] can be used to obtain the statistics of each entry of y. To do that, $N_{\mathrm{MC}}$ number of random vectors, $x_{n}$, $n=1,2,\ldots ,N_{\mathrm{MC}}$, are uniformly selected within the ranges provided in Table 1. Then, for each random vector/realization, $x_{n}$, a deterministic simulation is performed by a deterministic simulator (please refer to Section 2.4 for the deterministic simulator used in this study). The results of $N_{\mathrm{MC}}$ deterministic simulations, $y_{n}=F\left(x_{n}\right)$, $n=1,2,\ldots ,N_{\mathrm{MC}}$, are then used to compute the mean and variance of observables as

(1) $$\begin{array}{cc} E[y] & \approx \frac{1}{N_{\mathrm{MC}}}\sum_{n=1}^{N_{\mathrm{MC}}}y_{n} \end{array}$$

(2) $$\begin{array}{cc} \mathrm{Var}[y] & \approx \frac{1}{N_{\mathrm{MC}}}\sum_{n=1}^{N_{\mathrm{MC}}}y_{n}^{2}-{\left(E[y]\right)}^{2} \end{array}$$

where summation on the vectors is an element-wise sum, so that E[·] and Var[·] operators are considered to be acting on each entry of the vector y separately. Clearly, the traditional MC method is straightforward to implement. However, it requires a large number of random realizations/deterministic simulations to obtain reasonably accurate statistics since the convergence rate for the accuracy of the mean is $1/\sqrt{N_{\mathrm{MC}}}$ [25]. To this end, the brute-force application of the traditional MC is not computationally feasible for uncertainty quantification of the SAR induced by UHF MRI RF coils since each deterministic simulation performed by MARIE [23] is computationally costly. In addition to computing mean and variance, the traditional MC can be used to assess each random variable’s impact on the observable. This sensitivity assessment can be performed via Sobol indices [41], which quantify the contributions of individual random variables to the output variance. The $k^{th}$ random variable’s Sobol index, $S_{k}$, can be calculated via

(3) $$S_{k}={\mathrm{Var}}_{x^{k}}\left[y\right]/\mathrm{Var}\left[y\right]$$

Here, ${\mathrm{Var}}_{x^{k}}\left[\;\cdot \;\right]$ is the variance of the observable with respect to the random variable $x^{k}$, which can be computed by performing MC by only considering the $k^{th}$ random variable while fixing all remaining random variables to their corresponding nominal values in Table 1.

### 2.2. The HDMR Technique

The HDMR, a powerful surrogate modeling technique, approximates N−dimensional observables with lower dimensional component functions as

(4) $$y=F\left(x\right)=\sum_{v\subseteq \Omega }F_{v}\left(x^{v}\right)$$

where $\Omega =\left\{1,2,\ldots ,N\right\}$ represents the set of random variable indices, and v denotes the subset of Ω with cardinality $\left|v\right|\in \left\{0,1,2,\ldots ,N\right\}$. Therefore, $F_{v}\left(x^{v}\right)$ is called the |v|−th order component function with respect to a |v|−dimensional random input vector, $x^{v}$ [42]. Expanding HDMR in (4) yields

(5) $$\begin{array}{cc} F\left(x\right)=F_{0} & +\sum_{i}F_{i}\left(x^{i}\right)\\ \; & +\sum_{i<j}F_{ij}\left(x^{i},x^{j}\right)+\cdots +F_{\Omega }\left(x^{1},...,x^{N}\right) \end{array}$$

In (5), $F_{0}$ denotes the zeroth-order component function that remains a constant. $F_{i}\left(x^{i}\right)$ is the first-order component function modeling the influence of $x^{i}$ on the observable. The second-order component function, $F_{ij}\left(x^{i},x^{j}\right)$, delineates the combined impact of input random variables $x^{i}$ and $x^{j}$, while the remaining terms in the expansion are the higher-order component functions. When expanded, the number of component functions in HDMR expansion scales with $\sum_{k=0}^{N}N!/\left((N-k)!k!\right)$, which increase rapidly with increasing *N*. To this end, to reduce the substantial computational expenses associated with the component function generation, HDMR is often truncated at a low order in practice, while discarding higher-order terms [32]. In many practical problems, encompassing correlations up to the second order among input random variables is generally sufficient to accurately describe the input–output relationship [43]. Therefore, in this study, the HDMR expansion is retained at a maximum of second order as

(6) $$F\left(x\right)\approx F_{0}+\sum_{i}F_{i}\left(x^{i}\right)+\sum_{i<j}F_{ij}\left(x^{i},x^{j}\right)$$

This truncated expansion can be better explained via an example. Suppose y is a function of three random variables (N=3) with indices Ω={1,2,3}. The component functions in the HDMR expansion of y=F(x) up to |v|=2 can be shown as:

(7) $$\begin{array}{ccc} |v|=0,\; & v=⌀, & F_{0}\\ |v|=1, & v=\{1\}, & F_{1}\left(x^{1}\right)\\ |v|=1, & v=\{2\}, & F_{2}\left(x^{2}\right)\\ |v|=1, & v=\{2\}, & F_{3}\left(x^{3}\right)\\ |v|=2, & v=\{1,2\}, & F_{1,2}\left(x^{1},x^{2}\right)\\ |v|=2, & v=\{1,3\}, & F_{1,3}\left(x^{1},x^{3}\right)\\ |v|=2, & v=\{2,3\}, & F_{2,3}\left(x^{2},x^{3}\right) \end{array}$$

which can yield

(8) $$\begin{array}{cc} F(x) & \approx F_{0}+F_{1}\left(x^{1}\right)+F_{2}\left(x^{2}\right)+F_{3}\left(x^{3}\right)\\ & +F_{1,2}\left(x^{1},x^{2}\right)+F_{1,3}\left(x^{1},x^{3}\right)+F_{2,3}\left(x^{2},x^{3}\right) \end{array}$$

These component functions can be obtained by the CUT-HDMR strategy. In this strategy, the component functions are interpolated on the multidimensional cuts passing through a reference point. In particular, the first- and second-order component functions are interpolated on lines and planes passing through reference point $\bar{x}$ [42]. Then, the contributions from the lower-order component functions, $F_{u}\left(x^{u}\right)$, with indices u, which is the subset of the index set v of the component function, $F_{v}\left(x^{v}\right)$, are subtracted. In other words, the component functions, $F_{v}\left(x^{v}\right)$, are obtained as

(9) $$F_{v}\left(x^{v}\right)={\left.F(x)\right|}_{x=\bar{x}\backslash x^{v}}-\sum_{u\subset v}F_{u}\left(x^{u}\right)$$

where $x=\bar{x}\backslash x^{v}$ denotes the condition where random variables, whose indices do not belong to subset v, are set to their respective values at the reference point $\bar{x}$, typically set to the mean values of random variables. For the above example with N=3, the component functions in (7) become

(10) $$\begin{array}{cc} F_{0} & =F(\bar{x})\\ F_{1}\left(x^{1}\right) & =F\left(x^{1},{\bar{x}}^{2},{\bar{x}}^{3}\right)-F_{0}\\ F_{2}\left(x^{2}\right) & =F\left({\bar{x}}^{1},x^{2},{\bar{x}}^{3}\right)-F_{0}\\ F_{3}\left(x^{3}\right) & =F\left({\bar{x}}^{1},{\bar{x}}^{2},x^{3}\right)-F_{0}\\ F_{1,2}\left(x^{1},x^{2}\right) & =F\left(x^{1},x^{2},{\bar{x}}^{3}\right)-F_{0}-F_{1}-F_{2}\\ F_{1,3}\left(x^{1},x^{3}\right) & =F\left(x^{1},{\bar{x}}^{2},x^{3}\right)-F_{0}-F_{1}-F_{3}\\ F_{2,3}\left(x^{2},x^{3}\right) & =F\left({\bar{x}}^{1},x^{2},x^{3}\right)-F_{0}-F_{2}-F_{3} \end{array}$$

To construct the HDMR expansion, an iterative scheme is employed [32]. This scheme allows carefully selecting the component functions significantly contributing to the observable and omitting the insignificant ones. Thereby, the scheme requires a minimum number of component functions and minimal computational resources to construct the HDMR expansion. In particular, the iterative scheme starts from |v|=0 by computing the zeroth-order component function, $F_{0}$, which is the observable value at $\bar{x}$ [42]. Next, the scheme proceeds to the computation of $F_{i}\left(x^{i}\right)$ defined on the lines, intersecting $\bar{x}$. After the computation of all first-order component functions, the scheme computes the weights associated with each random variable (dimension) via

(11) $$\eta_{i}=\left|E\left[F_{i}\left(x^{i}\right)\right]/F_{0}\right|$$

**Figure 1:**
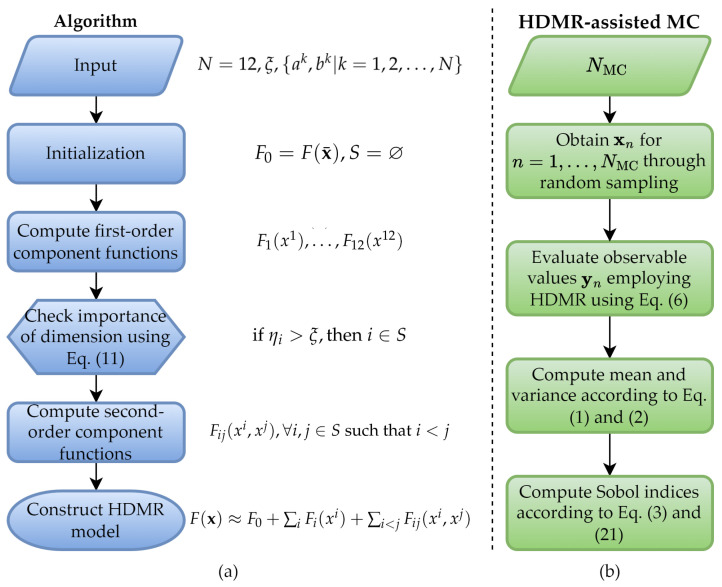
(**a**) Flowchart depicting the implementation of the truncated HDMR expansion applied in this study, with N=12. (**b**) Flowchart of HDMR-assisted MC method.

These weights, $\eta_{i}$, i=1,…,N, measure the sensitivity of a specific dimension of the input, x, to the observable, y, by comparing means of first-order component functions $E\left[F_{i}\left(x^{i}\right)\right]=\int F_{i}\left(x^{i}\right)dx^{i}$ to the mean of $F\left(\bar{x}\right)=F_{0}$. Therefore, when $\eta_{i}$ exceeds a prescribed tolerance, ξ, the random variable of that specific dimension is considered to have substantial contribution on y and is thus considered as “important” dimension. These indices are retained in set *S* for subsequent-level generation, ensuring only second-order functions satisfying v⊆S are incorporated when constructing HDMR. Figure 1a illustrates the flowchart of the construction of surrogate models using the HDMR technique.

For the above-given example (for N=3), assume that the indices of important dimensions are found to be S={2,3} after the computations of the zeroth- and first-order component functions and obtaining the weights $\eta_{i}$, i=1,2,3. Thus, the component function with indices {2,3} is included in the expansion while the other second-order component functions with indices {1,2} and {1,3} will not be computed, since at least one of their corresponding first-order terms is considered to be insignificant, with $\eta_{i}$ smaller than the tolerance, ξ. It should be noted that, after this construction, the indices of first-order component functions of insignificant dimensions are also included in set *S* since those first-order component functions are already computed. The component functions used to construct HDMR expansion are interpolated using the gPC expansion, explained next. After the HDMR model is constructed, statistics such as mean, variance, and sensitivity indices can be obtained, as shown in Figure 1b.

### 2.3. gPC Expansion

The fundamental principle of gPC involves determining the functional relationship between input vectors, $x^{v}$, and the component functions, $F_{v}\left(x^{v}\right)$. For simplicity in notation, let $\tilde{x}=\bar{x}\setminus x^{v}$ represent the scenario where the random variables with indices given in set v are retained, while all remaining random variables with indices out of set v are set to their mean values. To this end, $F\left(\tilde{x}\right)={\left.F(x)\right|}_{x=\bar{x}\setminus x^{v}}$ is the part in (9) to be approximated via gPC as

(12) $$F\left(\tilde{x}\right)\approx \sum_{n=0}^{N_{p}}\alpha_{n}\Phi_{n}\left(\tilde{x}\right)$$

where $\alpha_{n}$ are the coefficients to be calculated, and $N_{p}+1$ is the total number of terms in gPC expansion, such that $N_{p}=\left(|v|+p\right)!/\left(|v|!\;p!\right)-1$. Here, since the distribution of random variables is assumed to be uniform, $\Phi_{n}\left(\tilde{x}\right)$ is selected as the product of 1D Legendre polynomials [44] as

(13) $$\Phi_{n}\left(\tilde{x}\right)=\prod_{k\in v}\phi_{d_{n}^{k}}\left(x^{k}\right)$$

which denotes the joint polynomial basis functions of gPC, consisting of polynomials $\phi_{k}\left(x^{k}\right)$, which are individually defined for each random variable [44]. The multi-index $d_{n}^{k}$ satisfies $\sum_{k\in v}d_{n}^{k}⩽p$, such that the sum of degrees of all polynomials is constrained within a chosen degree, *p*. Also, when given degree *p*, all possibilities should be considered and incorporated when constructing (12). For example, for v={1,3}, |v|=2, and p=2, all possible polynomial bases are

(14) $$\begin{array}{c} \Phi_{0}\left(x^{1},x^{3}\right)=\phi_{0}\left(x^{1}\right)\phi_{0}\left(x^{3}\right)\\ \Phi_{1}\left(x^{1},x^{3}\right)=\phi_{1}\left(x^{1}\right)\phi_{0}\left(x^{3}\right)\\ \Phi_{2}\left(x^{1},x^{3}\right)=\phi_{0}\left(x^{1}\right)\phi_{1}\left(x^{3}\right)\\ \Phi_{3}\left(x^{1},x^{3}\right)=\phi_{1}\left(x^{1}\right)\phi_{1}\left(x^{3}\right)\\ \Phi_{4}\left(x^{1},x^{3}\right)=\phi_{2}\left(x^{1}\right)\phi_{0}\left(x^{3}\right)\\ \Phi_{5}\left(x^{1},x^{3}\right)=\phi_{0}\left(x^{1}\right)\phi_{2}\left(x^{3}\right) \end{array}$$

The coefficients $\alpha_{n}$ in (12) can be calculated by

(15) $$\alpha_{n}=\int_{\Omega }F\left(\tilde{x}\right)\Phi_{n}\left(\tilde{x}\right)d\tilde{x}$$

which is often difficult to compute analytically since analytical results of $F(\tilde{x})$ are typically elusive for complex nonlinear systems. Thereby, the tensor-product Gauss–Legendre (GL) quadrature integration rule [44] is implemented to calculate gPC coefficients as

(16) $$\alpha_{n}\approx \sum_{j=1}^{N_{\mathrm{GL}}^{\left|v\right|}}F\left({\tilde{x}}_{j}\right)\Phi_{n}\left({\tilde{x}}_{j}\right)w_{j}$$

Here, ${\tilde{x}}_{j}$ and $w_{j}$ are the collocation points and weights dictated by the GL quadrature rule, while the numbers of points and weights are ${\left(N_{\mathrm{GL}}\right)}^{\left|v\right|}$, where the cardinality of v, |v|, is the power of the number of GL collocation points selected along each dimension, denoted by $N_{\mathrm{GL}}$. Once these coefficients are computed, the component functions of HDMR can be approximated using the gPC method.

In short, the proposed methodology leverages the strengths of both techniques: HDMR’s ability to mitigate the curse of dimensionality and gPC’s capability to interpolate the component functions efficiently and accurately. Note that, while any univariate and bivariate interpolator could be employed, the application of gPC expansion on these subproblems demonstrates efficacy [42]. Furthermore, in determining the coefficients of each gPC expansion, the GL quadrature rule, delineated in (16), is applied. The observable values at collocation points of the GL quadrature are computed using a deterministic simulator, which is elaborated in the subsequent subsection. The number of required deterministic simulations corresponding to collocation points to construct HDMR can be calculated by:

(17) $$N_{\mathrm{cp}}=1+\sum_{v\subseteq S}\;{\left(N_{\mathrm{GL}}-1\right)}^{\left|v\right|}$$

where $N_{\mathrm{cp}}$ is the total number of collocation points and $N_{\mathrm{GL}}$ is kept fixed across all dimensions of univariate and bivariate component functions with indices, which are determined by the abovementioned iterative scheme and stored in the index set of *S*. In this study, we use odd-number GL quadrature rules, which share one collocation point (positioned at the reference point $\bar{x}$) for all component functions, while many collocation points of second-order component functions are already computed when interpolating the first-order component functions.

### 2.4. Deterministic Simulator MARIE [23]

To compute the component functions of HDMR expansion, the observable values on collocation points are computed via MARIE (MAgnetic Resonance Integral Equation suite) software [23], an open-source MATLAB-based simulator designed for fast EM analysis of MRI systems. The primary focus of MARIE is to offer comprehensive EM simulations in the context of the human body, targeting the determination of port parameters, E-field distribution, and key metrics like B1+, B1−, and local SAR. At its core, MARIE uses several integral equation methods. The inhomogeneous human body is voxelized and E-fields and currents inside the human body are solved using the volume integral equation technique. In parallel, the RF coils and shields, perfect electric conductors, are discretized by surface triangles, and the currents on them are computed using the surface integral equation technique. These techniques are carefully coupled, leveraging the volume–surface integral equation approach. Moreover, MARIE employs a fast iterative solution method for computational efficiency, incorporating a fast Fourier transform acceleration and special preconditioning technique for rapid iterative convergence. In this study, the observable values provided by MARIE are used to generate the surrogate models across voxels in the head model. The constructed surrogate models offer a computationally efficient representation for capturing the intricate relationships between the input random variables and the output MRI-induced E-field and SAR values in the head model.

## 3. Numerical Results and Discussion

This section demonstrates the proposed computational framework’s accuracy, efficiency, and applicability in obtaining the statistics of the UHF MRI RF coil-induced SAR on an MRI-derived head model. To construct the surrogate models, the tolerance for HDMR component function selection, ξ, is set to $10^{-2}$, while the number of GL quadrature points along each dimension, $N_{\mathrm{GL}}$, is set to 3, 5, or 7. As alluded in Section 2, the relative permittivities and conductivities of six types of head tissues are uniformly distributed in the ranges provided in Table 1, while the observables are the SAR values. To evaluate the accuracy of the surrogate models, the relative error of the observable on each voxel, $err_{i}$, $i=1,\ldots ,N_{\mathrm{vox}}$, is computed via

(18) $$err_{i}=\frac{1}{N_{\mathrm{test}}}\sum_{n=1}^{N_{\mathrm{vox}}}\left|\frac{y_{n}^{i}-{y_{n}^{i}}^{\prime }}{y_{n}^{i}}\right|$$

where $N_{\mathrm{test}}=100$ is the number of testing points, randomly selected according to the distributions shown in Table 1, while $y_{n}^{i}$ and ${y_{n}^{i}}^{\prime }$ represent the observable value on $i^{th}$ voxel obtained by the deterministic simulator and surrogate model, respectively. Once the accuracy of each surrogate model of each voxel is assessed via its associated relative error, the overall accuracy of all surrogate models is evaluated via

(19) $$\begin{array}{cc} err_{\max} & =\max_{i}\left\{err_{i}\right\},\;i=1,2,\ldots ,N_{\mathrm{vox}}\\ err_{\mathrm{ave}} & =\frac{1}{N_{\mathrm{vox}}}\sum_{i=1}^{N_{\mathrm{vox}}}err_{i} \end{array}$$

Here, the maximum relative error, $err_{\max}$, gives insight into worst-case scenarios or outlier behavior, whereas the average relative error, $err_{\mathrm{ave}}$, presents a more generalized view of the accuracy across the entire head model.

### 3.1. Numerical Settings

**Figure 2:**
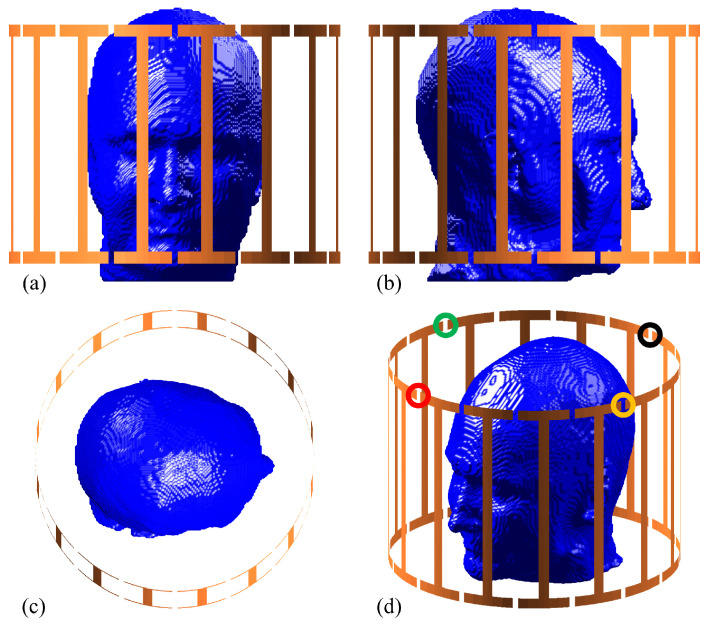
The MRI-derived head model in an MRI birdcage coil with the locations of activated ports highlighted. (**a**) Front view; (**b**) right side view; (**c**) top view; (**d**) port locations: port 1 (red), port 5 (green), port 9 (black), and port 13 (yellow).

In the considered MRI scenario, a birdcage RF coil provided in MARIE [23] is used to stimulate a human head model, as depicted in Figure 2. This 32-port birdcage coil with a 140 mm radius operates at 298.06 MHz for 7 T MRI scans and is initially excited from the port 1 [Figure 2d]. Thereafter, ports 5, 9, and 13, demonstrated in Figure 2d, are also activated to examine the accuracy of the constructed surrogate models, as discussed in subsequent subsections. The human head model is derived from an MR image selected from the OASIS2 dataset [45]. Initially, the MR image is transformed into a tetrahedral mesh using the headreco function [46] of SimNIBS [47]. This process yields a segmented head model with tissues, including white matter, grey matter, cerebrospinal fluid (CSF), bone, scalp, and eye humor. The conductivities and relative permittivities of these tissues are sequentially encapsulated in $x=[\varepsilon_{r1},\ldots ,\varepsilon_{r6},\sigma_{1},\ldots ,\sigma_{6}]$ and set randomly or according to the collocation point for each deterministic simulation. For compatibility with MARIE, the segmented head model is then converted to a voxel head model situated in a computational domain of 145×145×145 with a voxel size of 1.6 mm, where 889,850 voxels occupy tissues. The head model is positioned at the center of the computational domain so that the head model coincides with the birdcage coil’s center [Figure 2].

### 3.2. Accuracy

The accuracy of the surrogate models generated by the proposed method is examined. To this end, the surrogate models of the SAR for all voxels in the head model are obtained by two approaches. In the first approach, called the *direct approach*, the input–output relation is directly formed between the input vector of the head tissues’ dielectric properties, x, and the output vector storing all SAR values on all 889,850 tissue voxels. In the second approach, called the *indirect approach*, the input vector remains unchanged, while the components of E-fields, $\left[E_{x},E_{y},E_{z}\right]$, are considered as the output and stored in an output vector of a size of 2,669,550; each entry of the vector is a complex number. In the indirect approach, after the construction of the surrogate models of E-fields, those are used to compute the SAR values on each tissue voxel *i*, ${\mathrm{SAR}}_{i}$, via

(20) $${\mathrm{SAR}}_{i}=\left(E_{x,i}^{2}+E_{y,i}^{2}+E_{z,i}^{2}\right)\;\cdot \;\sigma_{i}/\rho_{i}$$

where $\left[E_{x,i},E_{y,i},E_{z,i}\right]$ are the components of E-fields on each voxel *i*. The density, $\rho_{i}$, stands consistent for each tissue type throughout the study, as shown in Table 1. The accuracy of the surrogate models obtained using direct and indirect approaches is assessed using the SAR values as observables in (18) and (19). To test the accuracy with different parameters, first, the HDMR expansion is truncated right after the first component functions, and $N_{\mathrm{GL}}$ is set to 3, obtaining gPC coefficients in (16). Furthermore, to achieve better accuracy, the number of component functions or GL points is increased in subsequent computations. This increase ensures a balanced approach between computational efficiency and the accuracy of the outcomes in the surrogate model assessments. Specifically, the number of GL points is augmented to 7 to improve approximation of component functions, or the HDMR is extended to encompass second-order component functions, which describe the combined effects between entries of the input vector. The results are shown in Table 2 for the direct approach and Table 3 for the indirect approach.

A straightforward comparison between Table 2 and Table 3 reveals that, generally, the results obtained through the indirect approach surpass those from the direct approach. This observation can be linked to the relationship between SAR values and the E-field, as shown in (20). The added complexity and interactions introduced by the squaring operations might necessitate a larger number of collocation points and the incorporation of higher-order terms to achieve a similar level of accuracy as that achieved when modeling indirectly.

Moreover, based on the data presented in the tables, it is noticeable that, when including solely the first-order component functions, there exists no direct correlation between an increment in the number of collocation points and the enhancement of accuracy in both scenarios. This suggests that the augmentation of collocation points does not significantly contribute to improving the accuracy of the surrogate modeling technique. Conversely, upon incorporating the second-order component functions, a notable enhancement in accuracy is observed, as evidenced by the substantial reduction in both mean and maximum relative errors. The optimal results are obtained in the second scenario, where the mean error is noted to be 0.26% and the maximum error is 1.65%, ensuring the confined error range for each voxel. In light of this, one should notice that the optimal accuracy in the study is attained in a scenario necessitating 1105 simulations. Considering the time-intensive nature of the deterministic simulations by MARIE, this approach might not be pragmatically viable. However, employing a mere 289 simulations yields results that, although slightly inferior to the 1105-simulation case in accuracy, offer a more feasible balance between accuracy and efficiency. Therefore, the subsequent analysis will be performed upon the 289-simulation case, utilizing a total order of 2 for component functions and three GL quadrature points along each dimension.

**Figure 3:**
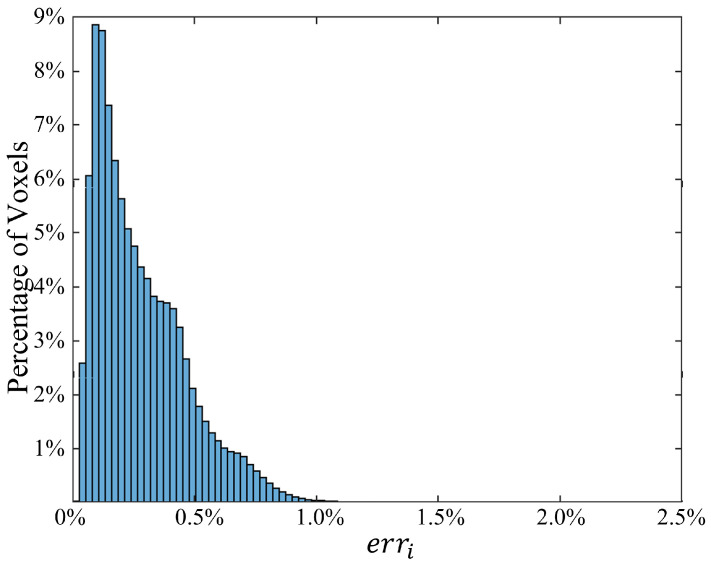
Relative error distributions for 889,850 tissue voxels. Derived from the second scenario where the total order for component functions is 2, with 3 GL quadrature points along each dimension.

**Figure 4:**
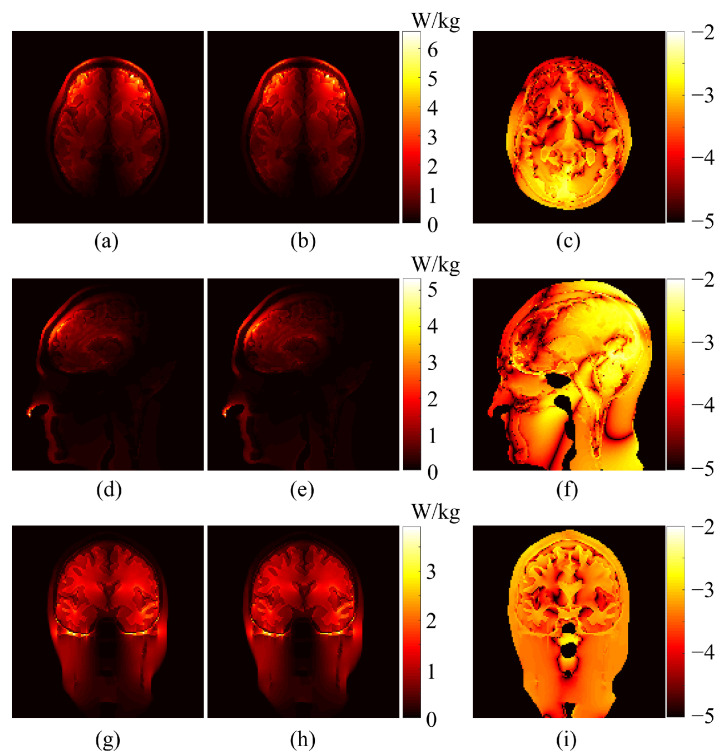
Comparison of the SAR on slices. The ground truth (**Left**), approximation via proposed framework (**Mid**), and the logarithm of the relative error between the ground truth and approximation (**Right**). (**a**) Ground truth of the axial slice. (**b**) Approximate SAR of the axial slice. (**c**) Logarithm of relative error between (**a**,**b**). (**d**) Ground truth of the sagittal slice. (**e**) Approximate SAR of the sagittal slice. (**f**) Logarithm of relative error between (**d**,**e**). (**g**) Ground truth of the coronal slice. (**h**) Approximate SAR of the coronal slice. (**i**) Logarithm of relative error between (**g**,**h**).

The distribution of voxel-based relative errors is shown in Figure 3. Indeed, most of the voxels exhibit an error of less than 1%, with only 0.339% of voxels surpassing this value. In Figure 4, selected slices are presented to highlight the accuracy of the proposed method under the second scenario. The left column depicts the ground truth SAR values obtained from the deterministic simulator MARIE, while the middle column displays values approximated by the HDMR-generated surrogate models. The right column illustrates the logarithm of the relative difference. With a logarithmic error spanning 2 to 5 digits, these illustrations underscore the high accuracy achieved by the proposed method.

The accuracy of the HDMR technique is assessed by comparing it with other surrogate modeling techniques such as RVFL [33,34], ELM [34,35], single-layer NN [36], GP [37], and least square-based gPC [38]. Each of these techniques is applied within the framework of two scenarios (direct and indirect) and their performances are assessed in terms of SAR values utilizing the same error metrics. To ensure a fair comparison, a training set of 300 points is generated through Latin Hypercube Sampling; the testing set remains the same, with 100 testing points. The results are shown in Table 4 and Table 5. Note that RVFL, ELM, and single-layer NN possess associated hyper-parameters. Therefore, multiple test cases are conducted to fine-tune these parameters, and the most optimal results are presented in the tables to ensure a fair and comprehensive comparison. As the tables illustrate, the proposed HDMR significantly outperforms all other surrogate modeling techniques.

The proposed technique is further examined under various conditions by individually activating other ports (Port 5, Port 9, and Port 13), demonstrating the reliability of the approach. All results, obtained under the second (‘indirect’) scenario, incorporating a total of second-order component functions and three GL points, are presented in Table 6. One can see that the mean relative errors for all conditions exhibit a consistent behavior, fluctuating within a narrow range. This indicates a stable performance of the proposed method, irrespective of the port activated. However, while the mean relative error demonstrates satisfactory performances, reflecting the method’s general effectiveness, the maximum relative error of port 9 is notably higher than that in all other conditions. The discrepancy in this case could be attributed to the spatial location of the voxels within an unexcited region. Given their substantial distance from the activation port, as shown in Figure 2d, the voxels near the nose region experienced less field excitation, resulting in comparatively lower SAR values. Thus, inconsequential deviations in prediction within this region can result in amplified maximum relative errors, which is expected.

### 3.3. Statistical Analysis

**Figure 5:**
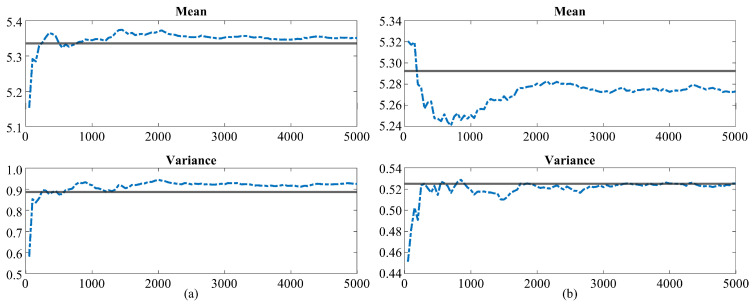
Convergence of mean (**top**) and variance (**bottom**) values for two different voxels, both computed using the 5000 point traditional MC method with increments of 50 random points/simulations. The black line represents the mean/variance values obtained via the HDMR-assisted MC method requiring 289 collocation points/simulations.

After establishing the accuracy of the proposed HDMR in the preceding section, it is important to assess the crucial statistical metrics obtained from the surrogate model. Employing the HDMR-assisted MC method with a sample size of 10,000 random points, the computed means and variances provide crucial insights into the performance and reliability of the proposed surrogate modeling technique. These statistical metrics are compared with the results derived from the traditional (brute-force) MC method, using simulations on MARIE with a sample size of 5000 randomly selected points. It is important to note that all points for the MC simulations are selected randomly, following the uniform distributions illustrated in Table 1. This comparative analysis is essential in corroborating the robustness and validity of the HDMR as a credible and efficient alternative to the traditional and computationally intensive MC methods for obtaining reliable statistical metrics. Figure 5 illustrates the convergence trends for the mean and variances of two selected voxels determined by the traditional MC method with increasing sample size. These metrics are juxtaposed and compared with the corresponding estimations from the proposed framework’s indirect approach. It becomes evident that the proposed method achieves a similar level of accuracy with a significantly lower number of deterministic simulations, underscoring its efficiency.

Furthermore, the maximum SAR values are also examined, given their paramount significance related to MRI safety regulations. This inquiry aims to discern the impacts of variations in tissue properties on the SAR values, which is vital for ensuring the safety of RF exposure. The nominal SAR values, obtained under nominal conditions of relative permittivities and conductivities, serve as a benchmark for comparison. The SAR values are assumed to follow normal distributions, which allow the estimation of maximum SAR values, calculated as the mean plus three times the standard deviation. These mean and standard deviation values are computed using HDMR-assisted MC methods, based on a sample of 10,000 random points.

In keeping with regulatory standards, voxel SAR values undergo conversion to 1g-SAR and 10g-SAR using a standard method. This process involves averaging the SAR values in a progressively expanding region of tissue-containing voxels surrounding a central voxel. The expansion continues one voxel at a time until the specified tissue mass, either 1 g or 10 g, is achieved [48]. This procedure is executed using the algorithm detailed in [49]. The analysis of the maximum SAR further underscores the importance of the uncertainties of input parameters, which inevitably contribute to the variances observed in SAR value distributions.

**Figure 6:**
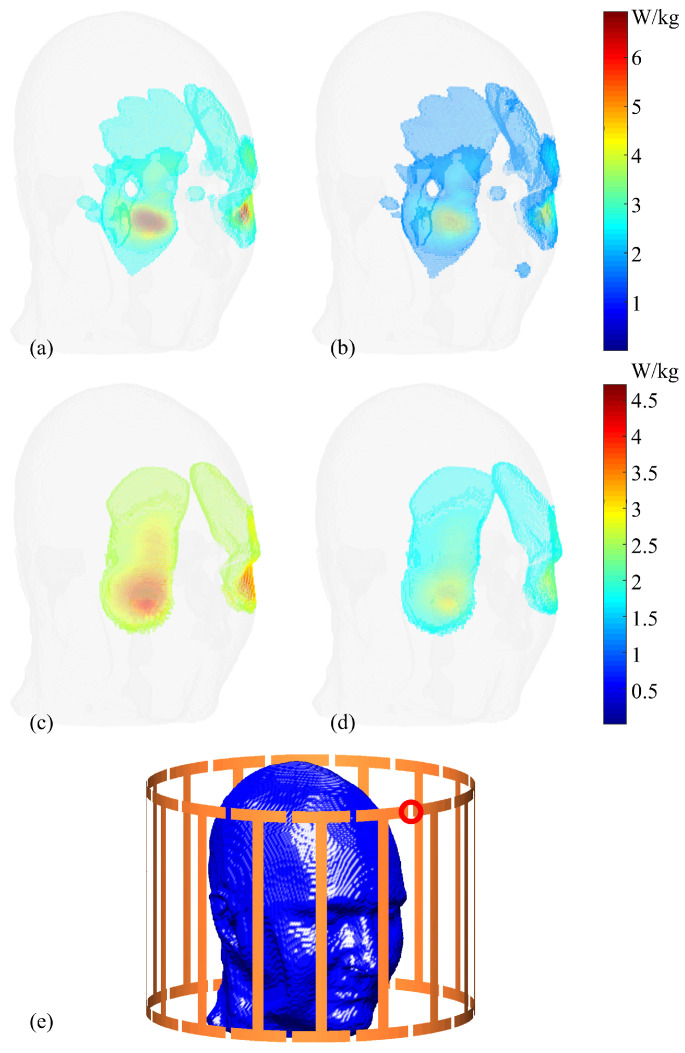
Comparison between maximum and nominal 1g-SAR and 10g-SAR distributions. For sub-figures (**a**–**d**), only the top 5% of voxels with highest SAR values are plotted. (**a**) Maximum 1g-SAR distributions. (**b**) Nominal 1g-SAR distributions. (**c**) Maximum 10g-SAR distributions. (**d**) Nominal 10g-SAR distributions. (**e**) Activation port location (circled in red).

Figure 6 presents a comparison between maximum and nominal 1g-SAR values. Note that only the top 5% of voxels with the highest values are selected for plotting to optimize visualization. Utilizing consistent colormaps, the voxels representing maximum values for the 1g-SAR exceed 6 W/kg, while those reflecting the nominal values for the same case are approximately 3.5 W/kg. For the 10g-SAR scenario, the maximum SAR values reach up to 4.5 W/kg, while voxels in the nominal values center around 2.5 W/kg.

**Figure 7:**
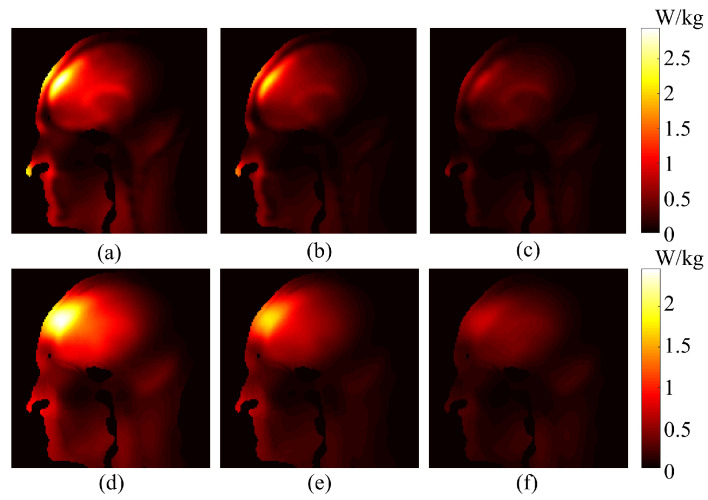
Comparison of sagittal slices between maximum and nominal SAR distributions, along with their differences. (**a**) Maximum 1g-SAR. (**b**) Nominal 1g-SAR. (**c**) Difference between (**a**,**b**). (**d**) Maximum 10g-SAR. (**e**) Nominal 10g-SAR. (**f**) Difference between (**d**,**e**).

Selected sagittal slices (Slice 72) of both 1g-SAR and 10g-SAR are depicted in Figure 7 for a comparative analysis between maximum and nominal SAR values, accompanied by their respective differences. For the 1g-SAR, there are noticeable increments of approximately 25% and 32% in the forehead and nose regions, respectively. Similarly, the 10g-SAR reveals an approximate 35% elevation in the forehead area.

### 3.4. Sobol Indices

For voxel *i*, its Sobol index with respect to v, $S_{v,i}$, can be computed as in (3) and then classified based on the type of tissue it represents. The average Sobol indices for a given tissue type *t*, denoted by $S_{v}^{t}$, is calculated by averaging $S_{v,i}$ across all voxels of the same tissue type, such that

(21) $$S_{v}^{t}=\frac{1}{N_{t}}\sum_{i=1}^{N_{t}}S_{v,i}$$

where $N_{t}$ indicates total number of voxels of tissue type *t*.

**Figure 8:**
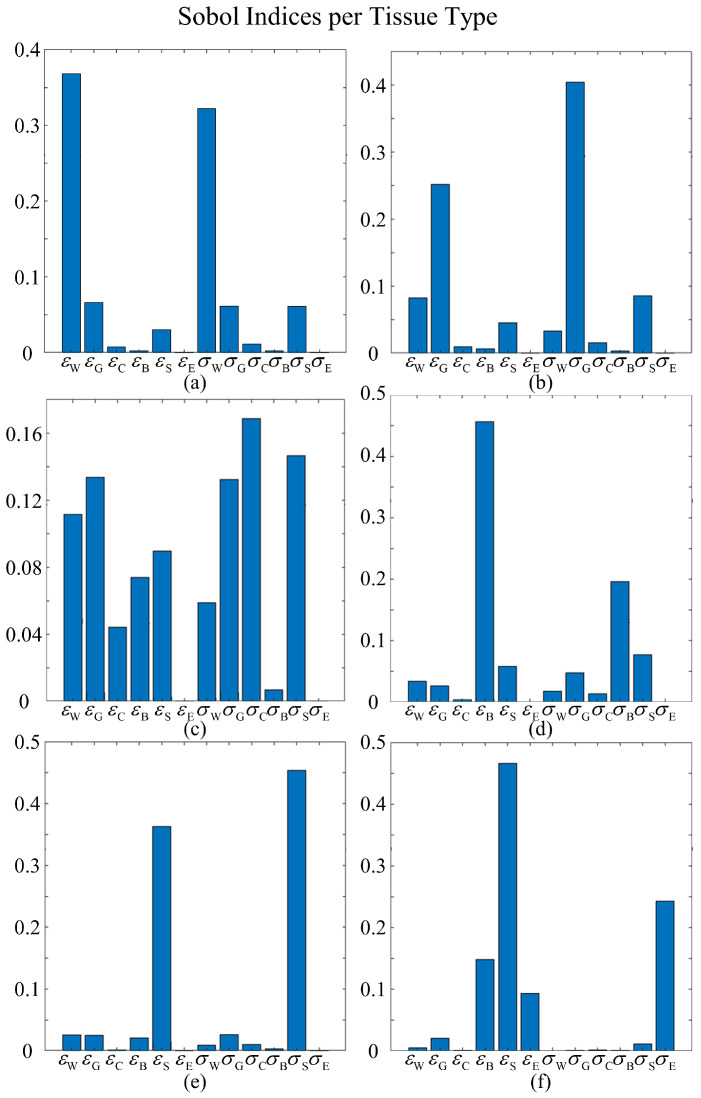
Averaged Sobol indices for each tissue type. The x-axis depicts input dimensions, where $\varepsilon_{r}$ is relative permittivity and σ is conductivity; W, G, C, B, S, E represents white matter, grey matter, CSF, bone, scalp, and eye humor, respectively. Sub-figures show Sobol indices for (**a**) white matter, (**b**) grey matter, (**c**) CSF, (**d**) bone, (**e**) scalp, and (**f**) eye humor.

Figure 8 displays the first-order Sobol indices of each tissue type *t* with respect to each input dimension. For most tissues, their own relative permittivity and conductivity have the greatest influence, followed closely by the relative permittivity and conductivity of neighboring tissue types. For the white matter, the primary influencers are its inherent relative permittivity and conductivity, with grey matter’s electrical properties coming next. In the case of grey matter, in addition to its own relative permittivity and conductivity, the relative permittivity of white matter and conductivity of the scalp also play significant roles. For CSF, while its own conductivity is the predominant contributor, other inputs also significantly influence the variances. This phenomenon can be attributed to the circumstance that CSF is a common neighbor to most tissues, resulting in its SAR values being impacted by the changes in the properties of adjacent tissues as well. This suggests a complex interaction between the CSF and its surrounding environment, emphasizing the necessity of incorporating second-order component functions in the HDMR framework. These functions are crucial for capturing the inputs’ combined effects, offering more accurate and detailed models. Sobol indices for bone and scalp tissues display similar patterns, with their respective relative permittivities and conductivities exerting the most impacts. The results for the eye require further analysis, given that the relative permittivity of the scalp appears to be the most influential dimension.

## 4. Conclusions

In this paper, a computational framework for uncertainty quantification of SAR and E-field values at voxel levels for MRI head scans at 7 T was proposed. This framework constructs the surrogate models for SAR distributions in head MRI scans utilizing the HDMR technique and then conducts statistical and sensitivity analyses on the observables. The application of the proposed framework to realistic head models demonstrated that the surrogate models are not only accurate in predicting SAR distributions but also significantly reduce the computational requirements compared with traditional MC methods. Subsequent statistical analysis revealed that 20% uncertainties in tissue dielectric properties could result in variations as substantial as 30% in the observed SAR values within certain regions. This highlights the importance of considering such uncertainties. The framework’s abilities to predict SAR distributions accurately and quantify the effects of variability in tissue properties underscore its potential as a valuable tool for supporting the analysis, design, and safety assessment of novel UHF MRI RF coils. Current research is focusing on the framework’s application with deep learning techniques for the SAR prediction on any provided head model [50]. The ultimate goal is to ensure the highest level of safety and efficacy in MRI procedures, particularly as the technology evolves and becomes more complex. This paper’s contribution represents a fundamental advance, providing a robust tool for researchers and clinicians in the rapidly advancing field of UHF MRI technology.

## Figures and Tables

**Table 1:** Variability in tissue dielectric properties.

Tissue	$\varepsilon_{r}$	Range	Symbol	RV	σ (S/m)	Range	Symbol	RV	ρ (kg/m^3^)
White Matter	43.8	[35.04, 52.56]	$\varepsilon_{r1}$	$x_{1}$	0.413	[0.33, 0.50]	$\sigma_{1}$	$x_{7}$	1041
Grey Matter	60.0	[48.00, 72.00]	$\varepsilon_{r2}$	$x_{2}$	0.692	[0.55, 0.83]	$\sigma_{2}$	$x_{8}$	1045
CSF	72.7	[58.16, 87.24]	$\varepsilon_{r3}$	$x_{3}$	2.220	[1.78, 2.66]	$\sigma_{3}$	$x_{9}$	1007
Bone	13.4	[10.72, 16.08]	$\varepsilon_{r4}$	$x_{4}$	0.083	[0.07, 0.10]	$\sigma_{4}$	$x_{10}$	1908
Scalp	49.8	[39.84, 59.76]	$\varepsilon_{r5}$	$x_{5}$	0.641	[0.51, 0.77]	$\sigma_{5}$	$x_{11}$	1109
Eye Humor	69.0	[55.20, 82.80]	$\varepsilon_{r6}$	$x_{6}$	1.520	[1.22, 1.82]	$\sigma_{6}$	$x_{12}$	1005

**Table 2:** Results of directly modeling SAR values.

Total Order of Component Functions	$N_{\mathrm{GL}}$	$N_{\mathrm{cp}}$	${\mathrm{err}}_{\max}$	${\mathrm{err}}_{\mathrm{ave}}$
1	3	25	34.36%	2.97%
1	5	49	33.80%	2.97%
1	7	73	33.74%	2.97%
2	3	289	14.28%	0.71%
2	5	1105	11.17%	0.51%

**Table 3:** Results of indirectly modeling SAR values through E-fields.

Total Order of Component Functions	$N_{\mathrm{GL}}$	$N_{\mathrm{cp}}$	${\mathrm{err}}_{\max}$	${\mathrm{err}}_{\mathrm{ave}}$
1	3	25	11.55%	1.46%
1	5	49	11.50%	1.48%
1	7	73	11.51%	1.47%
2	3	289	2.11%	0.28%
2	5	1105	1.65%	0.26%

**Table 4:** Results for other surrogate models of directly modeling SAR values.

Method	${\mathrm{err}}_{\max}$	${\mathrm{err}}_{\mathrm{ave}}$	Remarks
RVFL *	25.13%	3.35%	hidden nodes = 120
ELM *	27.00%	3.47%	hidden nodes = 120
Gaussian Process	85.64%	0.73%	/
Least Square PC	16.34%	0.77%	/
Single-layer NN *	≥100%	10.30%	nodes = 512
HDMR (proposed)	14.28%	0.71%	/

* For hyper-parameter tuning on RVFL and ELM, hidden nodes between 20 and 200 were tested, with an increment of 20 per step. Optimal results were observed at 120 for both ELM and RVFL. In the case of the single-layer neural network, tests were conducted evaluations spanning from 16 to 1024, progressing in powers of 2, with 512 yielding the optimal result. The optimal result is defined as the hyper-parameter that yields the lowest mean relative error on the testing set.

**Table 5:** Results for other surrogate models of indirectly modeling SAR values through E-fields.

Method	${\mathrm{err}}_{\max}$	${\mathrm{err}}_{\mathrm{ave}}$	Remarks
RVFL *	7.24%	1.27%	hidden nodes = 160
ELM *	7.90%	1.21%	hidden nodes = 140
Gaussian Process	16.41%	0.35%	/
Least Square PC	2.84%	0.41%	/
Single-layer NN *	45.85%	4.57%	nodes = 64
HDMR (proposed)	2.11%	0.28%	/

* For hyper-parameter tuning on RVFL and ELM, hidden nodes between 20 and 200 were tested, with an increment of 20 per step. Optimal results were observed at 160 and 140, respectively. In the case of the single-layer neural network, tests were conducted evaluations spanning from 16 to 128, progressing in powers of 2, with 64 yielding the optimal result. The optimal result is defined as the hyper-parameter that yields the lowest mean relative error on the testing set.

**Table 6:** Results for different activated ports using second-order component functions with three GL points.

Port No.	$N_{\mathrm{GL}}$	$N_{\mathrm{cp}}$	${\mathrm{err}}_{\max}$	${\mathrm{err}}_{\mathrm{ave}}$
Port 1	3	289	2.11%	0.28%
Port 5	3	289	2.08%	0.17%
Port 9	3	289	11.93%	0.31%
Port 13	3	289	3.82%	0.25%

## Data Availability

Data are made available with a granted proposal upon reasonable request to the authors.

## Appendix A. Nomenclature

*N*	Dimension of the input vector, N∈N
x	Input vector, $x\in R^{N}$
$x^{k}$	*k*-th element of the input vector, where k∈{1,2,…,N}
$\left[a^{k},b^{k}\right]$	Range of $x^{k}$
y	Observable vector
F(·)	Deterministic simulator mapping x to y
$N_{\mathrm{MC}}$	Number of MC simulations
$x_{n}$	*n*-th input vector of the simulation
$y_{n}$	*n*-th observable vector of the simulation
E[·]	Expected value operator
Var[·]	Variance operator
${\mathrm{Var}}_{x^{k}}\left[\;\cdot \;\right]$	Variance operator with respect to the random variable $x^{k}$
$S_{k}$	Sobol index for the *k*-th element of the input vector
Ω	The set of random variable indices, Ω={1,2,…,N}
v	Subset of Ω
|v|	Cardinality of v
$x^{v}$	Selection of the input vector x corresponding to the indices in v
$F_{0}$	Zeroth-order component function of HDMR
$F_{i}\left(x^{i}\right)$	First-order component function of HDMR
$F_{ij}\left(x^{i},x^{j}\right)$	Second-order component function of HDMR
$\bar{x}$	Reference point in CUT-HDMR
$\tilde{x}=\bar{x}\setminus x^{v}$	Input vector whose random variables indexed by set v are retained, and all others are set according to $\bar{x}$
α	Coefficients of gPC expansion
Φ(·)	Product of 1D Legendre polynomials
ϕ(·)	1D Legendre polynomials
$N_{\mathrm{GL}}$	Number of Gauss–Legendre quadrature points per dimension
$N_{\mathrm{CP}}$	Number of total collocation points
$N_{\mathrm{test}}$	Number of testing points
$err_{\max}$	Maximum error among all tissue voxels
$err_{\mathrm{ave}}$	Average error of all tissue voxels
